# Establishment of age group classification for risk stratification in glioma patients

**DOI:** 10.1186/s12883-020-01888-w

**Published:** 2020-08-20

**Authors:** Zhiying Lin, Runwei Yang, Kaishu Li, Guozhong Yi, Zhiyong Li, Jinglin Guo, Zhou Zhang, Peng Junxiang, Yawei Liu, Songtao Qi, Guanglong Huang

**Affiliations:** 1 Department of Neurosurgery, Nanfang Hospital, Southern Medical University, No. 1838 Guangzhou Avenue North, Guangzhou, 510515 Guangdong China; 2 The Laboratory for Precision Neurosurgery, Nanfang Hospital, Southern Medical University, Guangzhou, 510515 Guangdong China; 3 Nanfang Glioma Center, Guangzhou, 510515 Guangdong China

**Keywords:** Glioma, Age group classification, Risk stratification, Personalized treatment

## Abstract

**Background:**

Age is associated with the prognosis of glioma patients, but there is no uniform standard of age-group classification to evaluate the prognosis of glioma patients. In this study, we aimed to establish an age group classification for risk stratification in glioma patients.

**Methods:**

1502 patients diagnosed with gliomas at Nanfang Hospital between 2000 and 2018 were enrolled. The WHO grade of glioma was used as a dependent variable to evaluate the effect of age on risk stratification. The evaluation model was established by logistic regression, and the Akaike information criterion (AIC) value of the model was used to determine the optimal cutoff points for age-classification. The differences in gender, WHO grade, pathological subtype, tumor cell differentiation, tumor size, tumor location, and molecular markers between different age groups were analyzed. The molecular markers included GFAP, EMA, MGMT, P53, NeuN, Oligo2, EGFR, VEGF, IDH1, Ki-67, PR, CD3, H3K27M, TS, and 1p/19q status.

**Results:**

The proportion of men with glioma was higher than that of women with glioma (58.3% vs 41.7%). Analysis of age showed that appropriate classifications of age group were 0–14 years old (pediatric group), 15–47 years old (youth group), 48–63 years old (middle-aged group), and ≥ 64 years old (elderly group).The proportions of glioblastoma and large tumor size (4–6 cm) increased with age (*p* = 0.000, *p* = 0.018, respectively). Analysis of the pathological molecular markers across the four age groups showed that the proportion of patients with larger than 10% area of Ki-67 expression or positive PR expression increased with age (*p* = 0.000, *p* = 0.017, respectively).

**Conclusions:**

Appropriate classifications of the age group for risk stratification are 0–14 years old (pediatric group), 15–47 years old (young group), 48–63 years old (middle age group) and ≥ 64 years old (elderly group). This age group classification is effective in evaluating the risk of glioblastoma in glioma patients.

## Background

Over the past 30 years, the incidence of primary malignant brain tumors has increased at an annual rate of 1–2%, with an especially higher rate in the elderly population [1]. Glioma accounts for approximately 30% of all central nervous system (CNS) tumors and 80% of malignant primary brain tumors [2]. According to the 2016 World Health Organization (WHO) classification of tumors of the CNS, gliomas were classified into four grades (WHO grade I to IV) based on histologic criteria [3]. WHO grades I and II gliomas are recognized as low-grade gliomas (LGG) and grades III and IV are considered high-grade gliomas (HGG) [4]. In particular, glioblastoma (GBM, WHO grade IV) is the most common malignant tumor of the CNS, accounting for 45.2% of primary malignant the CNS tumors, and 54.0% of all gliomas [5]. The median survival of GBM patients is approximately 15 months, even after receiving multimodal therapies that include maximal surgical resection with the preservation of neurological functions, followed by adjuvant radiotherapy and chemotherapy [6].

Gliomas can occur at any age, with various incidences at different ages as reported in population-based studies [4, 7, 8]. LGG is the most common brain tumor in children, while HGG is the most frequent brain tumor in adults [9]. Tumors in the supratentorial areas of the brain (cerebral hemispheres and midline structures above the tentorium) were most frequent in adults, while subtentorial (brainstem and cerebellum) tumors were more common in young children than in adolescents and adults [10]. Besides, increasing studies have assessed age a prognostic factor. There are differences in prognosis among patients of different ages even with the same diagnosis. A single-center review of 70 patients with intracranial anaplastic oligodendroglioma showed that the median survival time of patients younger than 50 years old was significantly longer than that of patients older than 50 years old [11]. Other studies have shown that age was an important prognostic factor in addition to KPS score, surgical scope and histology [12, 13]. Therefore, for patients diagnosed with glioma by imaging examination and auxiliary examination, it is necessary to consider the age of the patients to perform personalized treatment for better outcomes.

However, there is no uniform age criterion for grouping glioma patients for personalized treatment [14]. Some glioma patient cohorts were divided into different age groups according to fixed age intervals [15], some were divided into two groups based on a certain age point [16], and others were divided based on the overall survival (OS) of the patients [17]. Different criteria for age grouping have led to inconsistent conclusions regarding the prognostic value of age. Some studies showed that age was not a prognostic factor in patients with glioma [18, 19]. while another population-based glioblastoma study with five age groups (< 50 years, 50–59 years, 60–69 years, 70–79 years, and > 80 years) showed that the OS of young patients (< 50 years) was significantly longer than that for elderly patients (> 50 years) (median 8.8 months vs 4.1 months, *p* < 0.001) [20]. Age-related studies involving a large number of glioma patients have yielded some relevant results [21, 22], but the age grouping criteria for these studies are influenced by several clinical factors, such as the tendency of clinical researchers. Therefore, there is an urgent need to establish a more appropriate age group classification criterion for better management of glioma patients.

For this purpose, we conducted a retrospective study collecting clinical data from 1502 patients with histologically proven gliomas in Nanfang Hospital between 2000 and 2018. Based on this cohort, we established a method of age group classification according to WHO grade for risk stratification in glioma patients and investigated the characteristics of different age groups in terms of gender, WHO grade, pathological subtype, tumor cell differentiation, tumor size, tumor location, and pathological molecular markers.

## Methods

### Data collection

A total of 1502 patients diagnosed with gliomas by pathological examination after surgery from 2000 to 2018 in Nanfang Hospital were enrolled in this study. The clinical data for age, gender, pathological diagnosis (according to the WHO 2000 Central nervous system tumor Classification), anatomic location of glioma, tumor size, and pathological molecular markers were collected (Supplementary Table S1).

The terminology of the anatomic location of glioma used in this study was based on the Central Brain Tumor Registry of the United States (CBTRUS), Brain and other Central Nervous System Tumor Site Groupings. We recognize that with the 2016 WHO classification of central nervous system tumors, many of the histological diagnostic criteria have undergone major changes and steps have been taken to align their histological grouping scheme with the 2016 WHO standards.

The pathological diagnosis included histological classification, WHO grade, and molecular expression. The pathological molecular markers included GFAP, EMA, MGMT, wt-P53, NeuN, Oligo2, EGFR, VEGF, IDH1, Ki-67, and ATRX. In addition, fluorescence in situ hybridization (FISH) detection of 1p/19q was also included. All pathological information was collected from the hospital medical records system.

### Calculation of age group cut-off points

Dummy variables were established by age groups of 1-I years old and I-82 years old (I: any age between 2 and 81). The established dummy variables were considered as independent variables, and a logistic regression model was established according to whether the patients were high-grade glioma or WHO IV grade glioma, which were set as dependent variables. The AIC was calculated to determine the best cut-off point for age among all models. The model with the lowest AIC value was regarded as the best model. The results showed that the diagnostic age classification criterion was 0–47 years old and ≥ 48 years old. The probability of high-grade glioma or WHO IV grade glioma in the age group ≥48 years old was greater than that in the age group 0–48 years old (78.4% vs 45.2, 50.2% vs 21.1%, respectively) (Supplementary Table S2).

Owing to the differences in the epidemiology between adults and pediatric glioma patients, the differences in surgical tolerance and treatment regimens between middle-aged and elderly patients, and the various prognoses of patients of different groups even with the same diagnosis, only two age groups for the classification of glioma patients were not sufficient in clinical practice. Therefore, these two groups were subdivided into four groups. First, dummy variables were created by age groups of 0-I years old and I-47 years old (I: any age between 2 and 46). The established dummy variables were considered as independent variables, and a logistic regression model was set up according to whether glioma patients were high grade glioma or WHO IV grade glioma. The AIC value for each model was calculated. The model with the smallest AIC value was regarded as the best model. According to whether the patient suffered from WHO IV glioma, the diagnostic age classification criteria were 0–14 years old (pediatric group) and 15–48 years old (young group). According to whether the patient was suffered from high-grade glioma, the diagnostic age classification criteria were 0–31 years old (pediatric group) and 31–48 years old (young group). The evidence suggests that the difference between the biological spectrum of the disease may be reflected in the diagnostic age, with the majority of the pediatric group belonging to the category described by Paugh et al. [23]. Although some of the molecular abnormalities encountered in HGG in children are reminiscent of secondary glioblastomas, these tumors rarely originate from existing LGGs [24]. Finally, 15 years old was chosen as the age for distinguishing the pediatric group from the adult group.

Second, dummy variables as independent variables were established by age groups of 48-I years old and ≥ I years old (I: any age between 49 and 80). The cut-off of the model with the minimum AIC value was calculated by the same method described above. The resulting diagnostic age classification criterion was 48–63 years old (middle-aged group) and ≥ 64 years old (elderly group). The probability of high-grade glioma or WHO IV grade glioma in the age group ≥64 years was greater than that of the age group 48–64 years old.

### Statistical analysis

The SPSS statistical software package (version 25, IBM Corp.) was used for all analyses. The statistical significance level was set as *p* < 0.05. Note that reported percentages may not add up to 100% due to rounding. Categorical variables are shown as numbers and percentages, while continuous variables are shown as the mean and standard deviation (SD). Pearson’s chi-square test was performed to compare the categorical data.

## Results

### Analysis of demographic and clinical characteristics

The study population comprised 875 (58.3%) male patients and 627 (41.7%) female patients. The ratio of males to females was 1.4:1. The age range was 1 to 82 years old and the mean age was 37.7 years old (SD = 17.7 years old). There were 137 (9.1%) patients were classified as WHO grade I, 530 (36.3%) patients were classified as WHO grade II, 381 (25.4%) patients were classified as WHO grade III, and 454 (30.2%) patients were classified as WHO grade IV (Supplementary Figure S2).

According to the 2016 WHO classification of tumors of the CNS, the 1502 glioma patients diagnosed and treated at Nanfang Hospital were subdivided into 23 histologically distinct types of primary glioma. Astrocytom as accounted for approximately 63.4% (*n* = 953) of all gliomas. The average diameter of glioma was 4.9 cm (SD = 2.0 cm). Gliomas mostly occurred in the frontal lobe (35.8%, *n* = 306) and temporal lobe (17.4%, *n* = 149). GBM represented the majority of gliomas (29.7%, *n* = 446). The distribution of tumor sites showed that 1396 (92.9%) cases occurred in the brain, 99 (6.6%) cases occurred in the spinal cord and cauda equina, and 7 (0.5%) cases involved the spinal cord, cauda equina, and brain. Detailed information for this cohort of glioma patients is recorded in Supplementary Table S1.

**Fig. 1:**
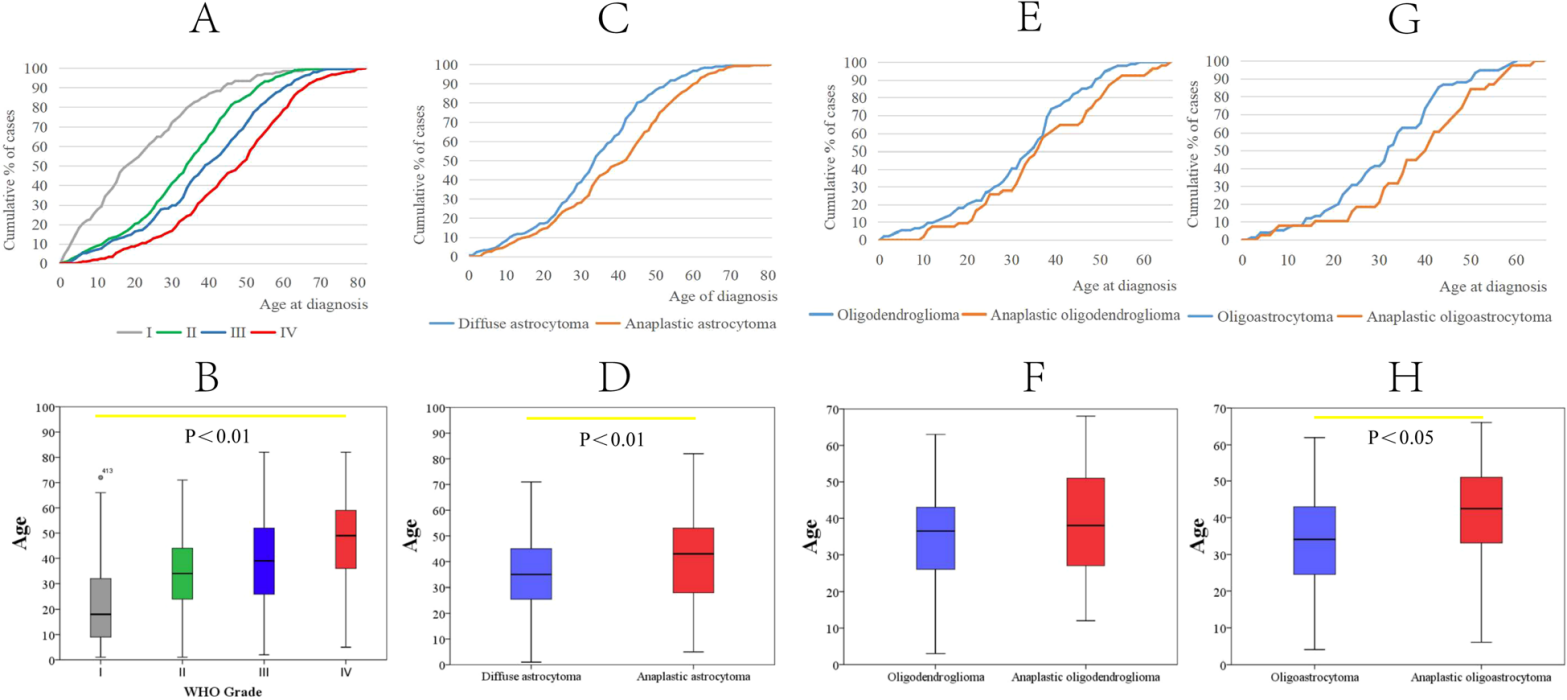
Cumulative age distribution and T test of the average age at diagnosis of different types of glioma. **a** Cumulative age distribution of WHO I-IV grade glioma, the mean age of glioma patients increases with the WHO grade (WHO I: 21.9 years, WHO II: 33.6 years, WHO III: 38.9 years and WHO IV: 46.3 years, respectively). **b** The diagnosed age boxplot figure of WHO I-IV grade glioma. **c** Cumulative age distribution of anaplastic astrocytoma and diffuse astrocytoma, there is likely for an earlier manifestation in diffuse astrocytoma. **d** The average age at diagnosis of anaplastic astrocytoma and diffuse astrocytoma. **e** Cumulative age distribution of Oligodendroglioma and anaplastic oligodendroglioma, most of oligodendroglioma and anaplastic oligodendroglioma arise in adults, with peak incidence in patients aged 30–50 years. **f** The diagnosed age boxplot figure of oligodendroglioma and anaplastic oligodendroglioma. **g** Cumulative age distribution of Oligoastrocytoma and anaplastic oligoastrocytoma, the median ages of patients with oligoastrocytoma are 34.0 years. The median age of patients with anaplastic oligoastrocytoma is 42.5 years. **h** The diagnosed age boxplot figure of oligoastrocytoma and anaplastic oligoastrocytoma

The median age at diagnosis for all primary glioma tumors was 38.0 years old. As shown by the cumulative curves of the proportion of gliomas across four WHO grades, gliomas of higher grades tended to be diagnosed at older ages (Fig. 1a, *p* < 0.05). The average age at diagnosis of WHO grade IV glioma was 46.3, while WHO grade I gliomas were diagnosed at 21.9 years, with an age gap of more than 24 years (Fig. 1b). The average ages at diagnosis of WHO grade II and III were 33.6 and 38.9 years, respectively (Fig. 1b). In addition, we compared the average age at diagnosis of various pathological subtypes of glioma. We found that anaplastic astrocytoma (WHO grade III) was diagnosed at an older age than that of individuals diagnosed with diffuse astrocytoma (WHO grade II) (Fig. 1c and d, 43.0 vs 35.0 years, respectively, *p* < 0.05). With a similar trend, anaplastic oligodendroglioma (WHO grade III) was diagnosed at a median age of 39.1 years, and oligodendroglioma (WHO grade II) was diagnosed at a median age of 34.8 years (Fig. 1e and f, *p* = 0.077). Besides, oligoastrocytoma (WHO grade II) and anaplastic oligoastrocytoma (WHO grade III) were diagnosed at average ages of 34.0 and 42.5 years, respectively (Fig. 1g and h, *p* < 0.05). Isocitrate dehydrogenase 1 (IDH1) is a vital marker for the molecular classification of glioma. In this cohort, when analyzing the average age at diagnosis of different IDH1 phenotypes by using the whole cohort, no significant differences were observed (Supplementary Figure S1B and D); however, IDH-wt GBM was diagnosed at an older age than that of individuals diagnosed with IDH-mut GBM (Supplementary Figure S1A and C, 49.3 vs 43.2, respectively, *p* < 0.05). These results indicated that the age at diagnosis was closely correlated with the WHO grade and pathological subtypes of glioma.

### Establishment of age group classification cut-off

**Fig. 2:**
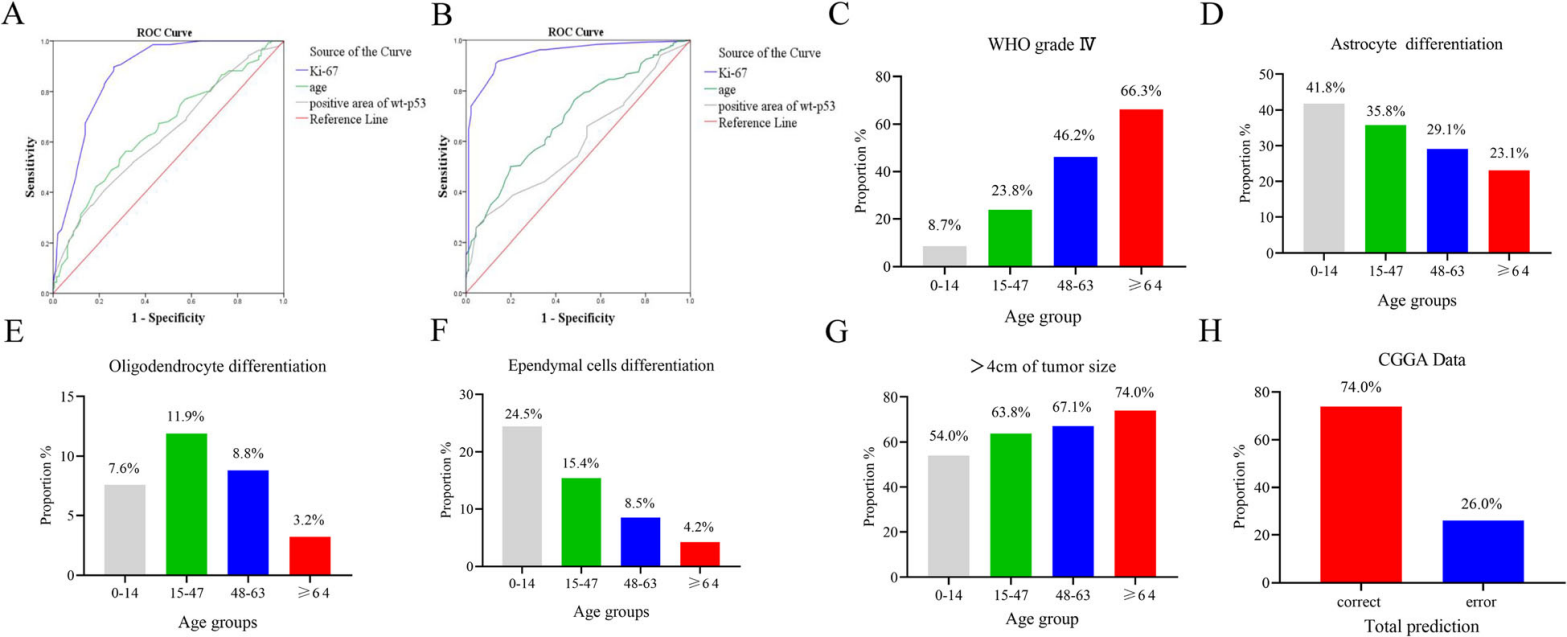
ROC curve of the sensitivity and specificity for diagnosing WHO IV glioma (**a**) and high grade glioma (**b**). Age, ki-67 and positive area of wt-p53 have great value for the diagnosis of WHO grade IV glioma and high-grade glioma. The proportion of WHO grade IV glioma (**c**), astrocyte differentiation (**d**), oligodendrocyte differentiation (**e**), ependymal cells differentiation (**f**) and >4 cm of tumor size (**g**) in four age groups. According to the discriminant classification of whether the pathological diagnosis of the patients was WHO grade IV or not, the prediction probability was taken as the discriminant dividing point, and the total judgment rate was 74.0% (**h**)

Age and positive area of Ki-67 and wt-P53 showed great value for the diagnosis of WHO grade IV glioma and high-grade glioma (Fig. 2a and b). The status of Ki-67 and P53 could be assessed only after surgery of biopsy, while the information of age could be obtained before surgery. Therefore, age could be an earlier factor for the evaluation of patients in clinical practice. We then sought to establish an age group classification for better management of patients according to the AIC method mentioned in the section of “method”. Glioma patients were divided into four age groups: 0–14 years old (pediatric group), 15–47 years old (youth group), 48–63 years old (middle-aged group) and ≥ 64 years old (elderly group). 12.3% of patients were 0–14 years old (pediatric group), 56.3% were 15–47 years old (middle-aged group), 25.1% were 48–63 years old (youth group), and 6.3% were ≥ 64 years old (elderly group). The proportion of primary WHO grade IV gliomas and larger tumor sizes (larger than 4 cm) increased with age (Fig. 2c and g), however, the proportions of glioma of astrocyte differentiation (only include WHO grade I-III) and ependymal cells differentiation decreased with age (Fig. 2d and f). Most of the gliomas of oligodendrocyte differentiation were found in 15–47 age group (Fig. 2e).

To examine the value of this age group classification in risk stratification of GBM, we collected data from 650 patients in the Chinese Glioma Genome Atlas (CGGA) database and calculate the proportion of different glioma grade in four age groups, respectively. The sensitivity of predicting WHO grade IV was 64.4%, the specificity was 79.1%, and the total judgment rate was 74.0% (*p* < 0.001) (Fig. 2h).

### Analysis of the pathological subtypes of glioma across four age groups

**Fig. 3:**
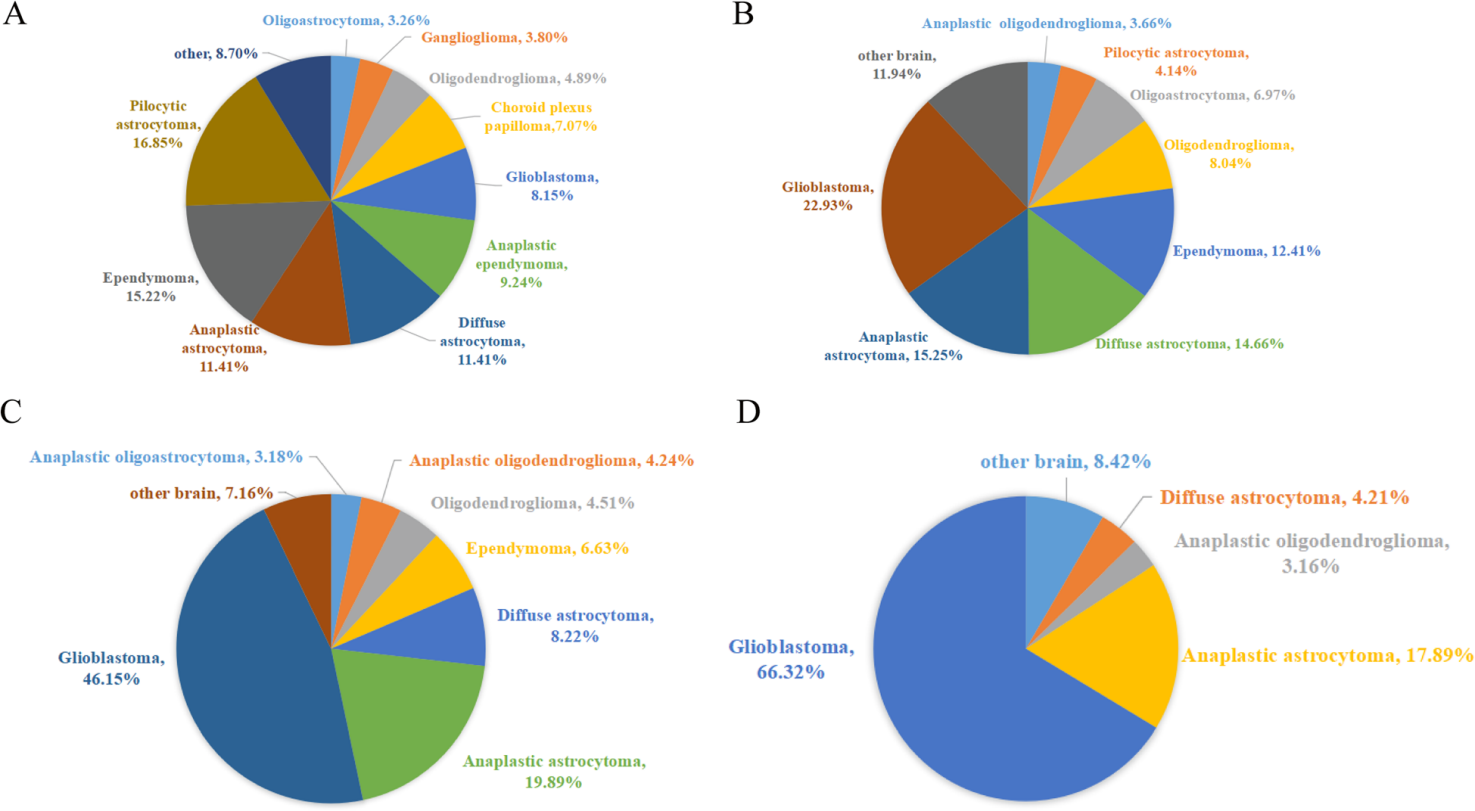
Histological distribution by Age groups. **a** Histological distribution by 0–14 years old group. **b** Histological distribution by 15–47 years old group. **c** Histological distribution by 48–63 years old group, and **d** Histological distribution by ≥64 years old group. In the 0–15 age group. The proportion of pilocytic astrocytoma in the histological distribution was 16.9%, however, glioblastoma accounted for the largest proportion of the age group 15–48 years old, 48–64 years old and ≥ 64 years old, with 22.9, 46.2 and 66.3% respectively

**Fig. 4:**
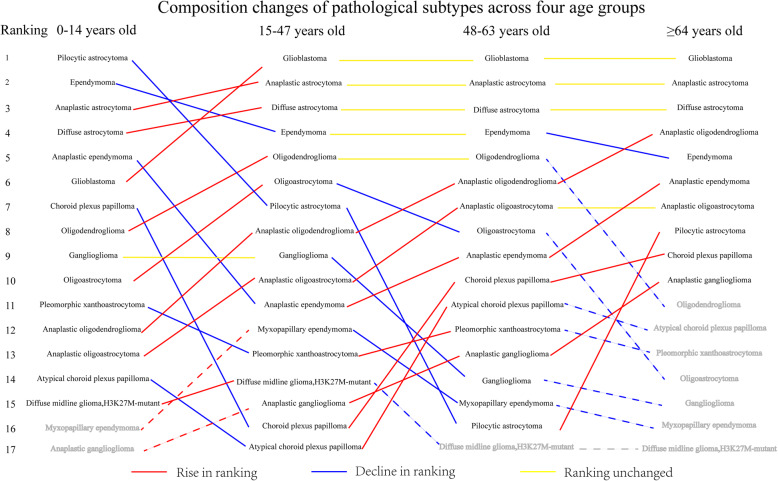
Composition changes of pathological subtypes across four age groups

In the pediatric group, the proportion of pilocytic astrocytoma was 16.9%, while GBM accounted for the largest proportion in the youth group, middle-age group and elderly group (22.9, 46.2 and 66.3%, respectively) (Fig. 3 and Supplementary Figure S3). Pilocytic astrocytoma, pleomorphic xanthoastrocytoma, ependymoma, anaplastic ependymoma, choroid plexus papilloma, atypical choroid plexus papilloma and ganglioglioma are predisposed to patients in pediatric group. Diffuse astrocytoma, diffuse midline glioma, H3K27M-mutant glioma, oligodendroglioma, oligoastrocytoma and myxopapillary ependymoma commonly occurred in youth group. Anaplastic astrocytoma, anaplastic oligodendroglioma and anaplastic oligoastrocytoma were more likely to occur in middle-age group. GBM and anaplastic ganglioglioma were more likely to occur in elderly group (p<0.001). The proportions of anaplastic oligodendroglioma and anaplastic ganglioglioma increased with age. Ependymoma gradually decreased in the younger age groups (Fig. 4).

### Analysis of glioma cell differentiation, size, and anatomic location across four age groups

Patients aged ≥64 years old were predisposed to gliomas of astrocyte differentiation. Patients aged 15–47 years old were predisposed to gliomas of oligodendrocyte and hybrid cell differentiation. Patients aged 0–14 years old were predisposed to gliomas of ependymal cell and other cells differentiation (Supplementary Table S2, *p* = 0.002). The proportion of tumors with sizes of 0–4 cm decreased with age; however, the proportion of tumors with sizes ranging from 4 to 6 cm was larger in older groups (Supplementary Table S2, *p* = 0.018).

In the pediatric group, the common locations of gliomas were the cerebellum and ventricle, accounting for 18.6 and 23.3%, respectively (Supplementary Table S3). However, in the youth and middle-age groups, the frontal lobe accounted for the largest proportion (Supplementary Table S3, *p* = 0.000). In the elderly group, the proportion of tumors in the frontal lobe and temporal lobe was higher than that in the other locations (Supplementary Table S3, 32.7 and 40.8%, respectively).

### Analysis of molecular marker expression in four age groups

**Fig. 5:**
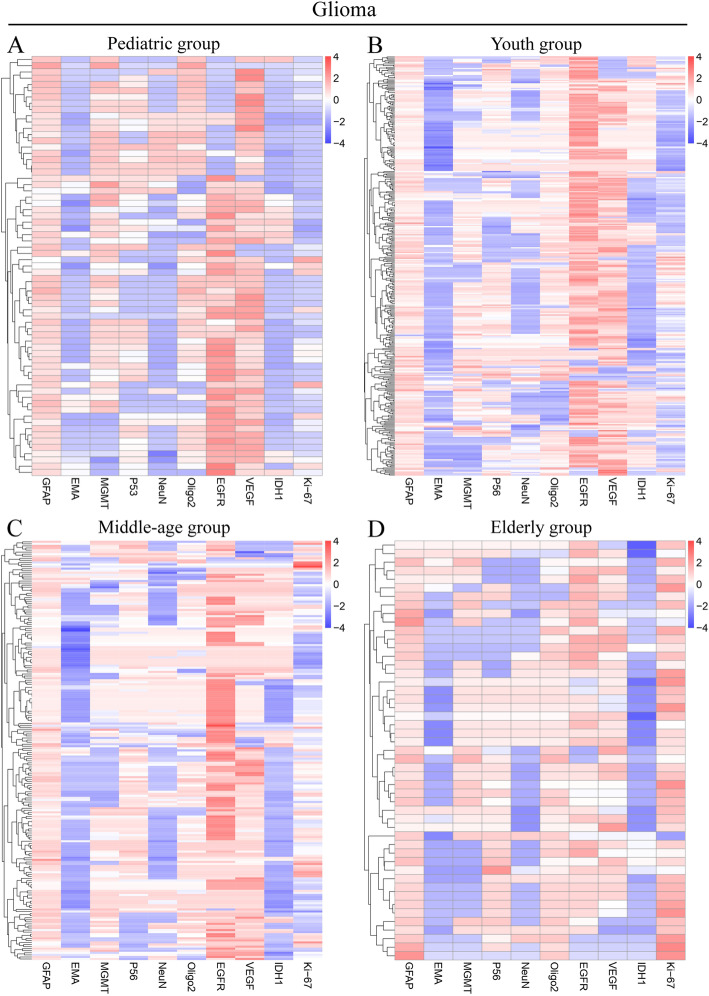
The glioma heatmap of 10-gene signatures by gene expression subtype. Representative genes are shown for each subtype. **a** Heatmap of pediatric group. **b** Heatmap of youth group. **c** Heatmap of middle-age group. **d** Heatmap of elderly group

The proportion of positive expression of glial fibrillary acidic protein (GFAP) was more than 90% in all age groups. Detailed information is recorded in Supplementary Table S2. The proportion of positive expression of IDH1-wt, Ki-67 and Oligodendrocyte transcription factor 2 (Oligo2) increased with age. The proportion of positive expression of epithelial membrane antigen (EMA), vascular endothelial growth factor (VEGF) and O-6-methylguanine-DNA methyltransferase (MGMT) were maximal in the pediatric group, while the proportion of positive expression of neuronal nuclei (NeuN) and epidermal growth factor receptor (EGFR) were highest in the middle-age group (Fig. 5). Besides, we analyzed the expression of glioma-associated genes in homogeneous groups, including subgroups of different cell origins, and different molecular subtypes, such as EGFR-positive and EGFR-negative gliomas. The results revealed great heterogeneity across the four age groups (Supplementary Figure S4, S5, S6, S7, S8, S9, S10, S11, Supplementary Table S4-S5).

## Discussion

Clinical and biological data clearly indicate that the characteristics and outcomes of malignant gliomas differ significantly between adults and children [9]. A number of studies have showed that the tumor-prone locations, histopathology, prognosis and some molecular markers are different in glioma patients of different ages [25, 26]. Growing research has shown that the molecular characteristics of GBM in elderly patients are more aggressive than those in young patients [27]. Childhood GBM displayed (on average) considerably fewer DNA copy number changes than histologically similar adult tumor [14–16]. In addition, the prognosis of glioma is particularly severe in older adults [26, 28]. The clinical practice patterns show that with increasing age, the application of surgical resection, radiotherapy and chemotherapy decreases [29–31]. Nevertheless, some elderly patients with glioblastoma can benefit from these therapies [30]. These elderly patients will receive aggressive treatment with radiation or chemotherapy. When considering treatment options for children with gliomas, neurosurgeons will try to avoid the deleterious effects of radiotherapy on the developing brains of children. Minimal dysfunction resulting from glioma and treatment should be achieved as much as possible with the expectation of children living to adulthood [32]. Moreover, age is regarded as an important factor related to the prognosis of glioma patients. Therefore, for patients diagnosed with glioma, age should be taken into consideration to perform personalized treatment for a better outcome. However, the criterion for appropriately dividing age groups of glioma patients remains an unresolved clinical problem.

A large number of studies used different age groupings, and these studies led us to differential conclusions about the prognosis value of age in glioma patients [18, 19, 33]. These contradictory conclusions could be partly explained by the difference in age classification criteria between different studies. In one study, a multivariate Cox regression model with different cutoff points was used to analyze the effect of age on OS, but only three age groups were compared, and univariate analysis was performed using prognostic factors as a classification criterion [17]. OS is a good indicator for evaluating patient outcomes, but confounding factors such as tumor size, tumor location, surgical resection extent, and patient compliance, might impair the accuracy of the relationship between age and OS.

To avoid the disturbance of confounding factors as much as possible, our study used the WHO grade of glioma as a dependent variable to assess the prognosis of glioma patients. The classification criteria for glioma patients based on age were 0–14 years old (pediatric group) and 15–47 years old (youth group), 48–63 years old (middle-aged group) and ≥ 64 years old (elderly group). This age group classification can be used for preliminary evaluation of newly-diagnosed glioma patients, and helps to perform precise management in clinical practice according to age group. Besides, we found that EGFR-positive expression was more common in the middle-age group, and the EGFR expression in IDH1-mut gliomas was more apparent. Therefore, patients with IDH1-mut glioma aged 48–63 years old might benefit from EGFR inhibitor therapy. Based on this age group classification, we further analyzed the characteristics of WHO grade, tumor size, tumor histology, and anatomical location among the four age groups. We found that the proportion of WHO grade IV gliomas and positive expression of Ki-67, Oligo2, and IDH-wt increased significantly in elderly age groups. In addition, in the older age group, more patients suffered from a heavy tumor burden (tumor size > 4 cm). Regarding the histology of glioma, pilocytic astrocytoma is the most common in children, while glioblastoma accounts for the largest proportion of adult groups. Many studies have demonstrated that patients with a higher grade of glioma have a worse outcome [6]. Moreover, a larger tumor burden might cause a higher risk of functional deficits, including motor dysfunction, impaired communication ability or decline in neurocognitive function [2]. Therefore, the prognosis of patients with gliomas can initially be evaluated according to age. On the other hand, patients grouping according to age has been widely used in clinical studies, but there is no uniform standard of age group classification for patients with glioma. The age group established on the basis of objective pathological diagnosis in this study will be helpful for clinical trials design in the future.

Glioma, especially glioblastoma, is a highly heterogeneous malignancy. In addition to the marked heterogeneity of tumor size and histopathology, the heterogeneity of the molecular characteristics of tumors is becoming increasingly important and is reported in several studies. According to the 2016 WHO classification, glioma is first classified according to histological features, and then more subtypes are classified according to molecular characteristics. There are a variety of indicators that are widely used in clinical practice (such as GFAP, EMA, MGMT, P53, NeuN, Oligo2, EGFR, VEGF, IDH1, Ki-67, 1p/19q), and these indicators are highly correlated with the prognosis of the patients [34–36]. Age-dependent occurrence and the effects of different biological markers have been reported in malignancies [37]. For example, the association between age and tumor grade, Ki-67 markers, apoptosis index, EGFR expression and erbB-2 expression has been reported in breast cancer [38]. A study indicated that the prognostic effects of P53, 1p, and *CDKN2A/p16* alterations are dependent on patient age [39]. Increasing translational studies have significantly advanced the understanding of glioma pathogenesis and have identified several prognostic factors. Higher tumor grade, older age [33], and increased expression of molecular biomarkers such as P53 [40], MGMT [41], PR [42], IDH1-wildtype [43], H3K27M mutation of pediatric HGG [44, 45], and Ki-67 [46], were related to poorer prognoses. Analysis of the pathological molecular markers across four age groups showed that the proportion of patients with larger than 10% area of Ki-67 positive expression or PR positive expression increased with age. Other molecular markers (GFAP, EMA, NeuN, EGFR, IDH1, CD3, and H3K27M) showed great heterogeneity among the four age groups.

Gender, age, anatomic location of the tumor, size of tumor and molecular markers are simple and objective parameters that can be collected easily in clinical practice or clinical studies on patients with glioma. Our research can provide clinicians with a simple method to evaluate the prognosis of glioma patients and help to promote the personalized management of glioma patients. In addition, for some clinical trials that need to divide participants of glioma into different groups, this age group classification based on WHO grade will be more objective. However, this study was limited by the sample size, and these data were retrospective. Hospital-based retrospective studies may lead to certain selection biases. Another limitation of this study was that we did not include patients with postoperative recurrence. Further validation of our results will require multicenter prospective studies with larger sample sizes.

## Conclusion

Our research indicated that the classification criteria based on the age for glioma patients were 0–14 years old (pediatric group), 15–47 years old (youth group), 48–63 years old (middle-aged group) and ≥ 64 years old (elderly group). Our cohort indicates that pilocytic astrocytoma accounts for the largest proportion in the 0–14 year age group, while GBM accounts for the largest proportion in the other three age groups. Besides, the proportion of tumors of 4–6 cm in size or with Ki-67 > 10% increases with WHO grade. This age group classification will help to improve the diagnosis, personalized treatment, and clinical trial design involved patients with glioma.

## Supplementary information

**Additional file 1: Figure S1.** Cumulative age distribution and T test of the average age at diagnosis of glioma. **A:** Cumulative age distribution of IDH1-wt glioma and IDH1-mut glioma. **B:** Cumulative age distribution of IDH1-wt glioma and IDH1-mut glioma. **C:** The diagnosed age boxplot figure of IDH1-wt GBM and IDH1-mut GBM. **D:** The diagnosed age boxplot figure of IDH1-wt GBM and IDH1-mut GBM.

**Additional file 2: Figure S2.** Constituent ratios of four age groups. The proportion of patients in the four age groups 0–14, 15–47, 48–63 and ≥ 64 years old.

**Additional file 3: Figure S3.** Distribution by Age groups of other histology. **A:** 0–14 years old. **B:** 15–47 years old. **C:** 48–63 years old. **D:** ≥64 years old. (subependymal giant cell astrocytoma, subependymoma, angiocentric glioma, chordoid glioma of the third ventricle, anaplastic ganglioglioma, desmoplastic infantile astrocytoma and ganglioglioma were not analyzed because the total number of patients was no more than three).

**Additional file 4: Figure S4.** Heatmap of 10-gene signatures by gene expression subtype. Representative genes are shown for each subtype. **A:** Heatmap of all glioma. **B:** Heatmap of all GBM. **C:** Heatmap of pediatric group. **D:** Heatmap of youth old group. **E:** Heatmap of middle-age group. **F:** Heatmap of elderly group.

**Additional file 5: Figure S5.** The heatmap of glioma derived from astrocyte differentiation (only including WHO grade I- III). **A:** Heatmap of pediatric group. **B:** Heatmap of youth group. **C:** Heatmap of middle-age group. **D:** Heatmap of elderly group.

**Additional file 6: Figure S6.** The heatmap of glioma derived from oligodendrocyte differentiation. **A:** Heatmap of pediatric group. **B:** Heatmap of youth group. **C:** Heatmap of middle-age group. **D:** Heatmap of elderly group.

**Additional file 7: Figure S7.** The heatmap of glioma derived from ependymal cells differentiation. **A:** Heatmap of pediatric group. **B:** Heatmap of youth group. **C:** Heatmap of middle-age group. **D:** Heatmap of elderly group.

**Additional file 8: Figure S8.** The heatmap of glioma with EGFR positive expression. **A:** Heatmap of pediatric group. **B:** Heatmap of youth group. **C:** Heatmap of middle-age group. **D:** Heatmap of elderly group.

**Additional file 9: Figure S9.** The heatmap of glioma with EGFR negative expression. **A:** Heatmap of pediatric group. **B:** Heatmap of youth group. **C:** Heatmap of middle-age group. **D:** Heatmap of elderly group.

**Additional file 10: Figure S10.** The heatmap of IDH1-mut glioma. **A:** Heatmap of pediatric group. **B:** Heatmap of youth group. **C:** Heatmap of middle-age group.

**Additional file 11: Figure S11.** The heatmap of IDH1-wt glioma. **A:** Heatmap of pediatric group. **B:** Heatmap of youth group. **C:** Heatmap of middle-age group.

**Additional file 12: Table S1.** Clinical and molecular characteristics of patients with gliomas (*n* = 1502).

**Additional file 13: Table S2.** Clinical and molecular characteristics of the patients with gliomas classified by age groups.

**Additional file 14: Table S3.** Anatomic location of gliomas classified by age groups.

**Additional file 15: Table S4.** Molecular characteristics of the patients with IDH1-mut gliomas classified by age groups.

**Additional file 16: Table S5.** Molecular characteristics of the patients with IDH1-wt gliomas classified by age groups.

## Data Availability

All data generated and analysed in this study are included in this article and supplementary materials.

## Abbreviations

AIC: Akaike information criterion
CNS: Central nervous system
WHO: World Health Organization
LGG: Low-grade gliomas
HGG: High-grade gliomas
GBM: Glioblastoma
OS: Overall survival
CBTRUS: Central Brain Tumor Registry of the United States
CGGA database: Chinese Glioma Genome Atlas database
FISH: Fluorescence in situ hybridization
SD: Standard deviation
IDH1: Isocitrate dehydrogenase 1
IDH-wt: IDH-wild type
GFAP: Glial fibrillary acidic protein
PR: Progesterone receptor
TS: Thymidylate synthase
Oligo2: Oligodendrocyte transcription factor 2
EMA: Epithelial membrane antigen
MGMT: O-6-methylguanine-DNA methyltransferase
NeuN: Neuronal nuclei
EGFR: Epidermal growth factor receptor
