# Supplementary figures and images for: Establishment of age group classification for risk stratification in glioma patients
Source: BMC Neurol. 2020 Aug 20;20:310. doi: 10.1186/s12883-020-01888-w (PMC7439690; doi:10.1186/s12883-020-01888-w)

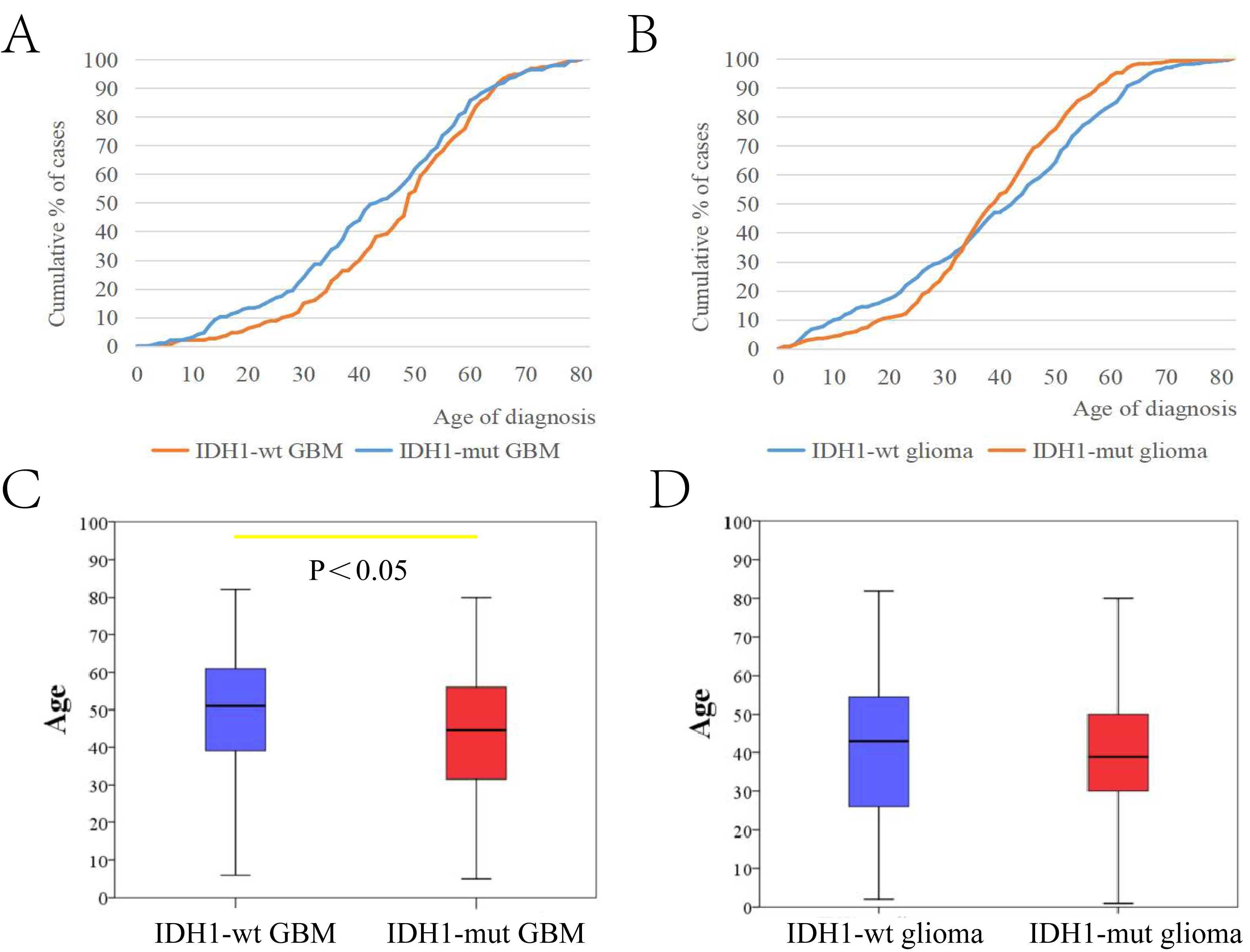

Supplement: Supplementary file 1 — Additional file 1: Figure S1. Cumulative age distribution and T test of the average age at diagnosis of glioma. A: Cumulative age distribution of IDH1-wt glioma and IDH1-mut glioma. B: Cumulative age distribution of IDH1-wt glioma and IDH1-mut glioma. C: The diagnosed age boxplot figure of IDH1-wt GBM and IDH1-mut GBM. D: The diagnosed age boxplot figure of IDH1-wt GBM and IDH1-mut GBM. [file 12883_2020_1888_MOESM1_ESM.tif]

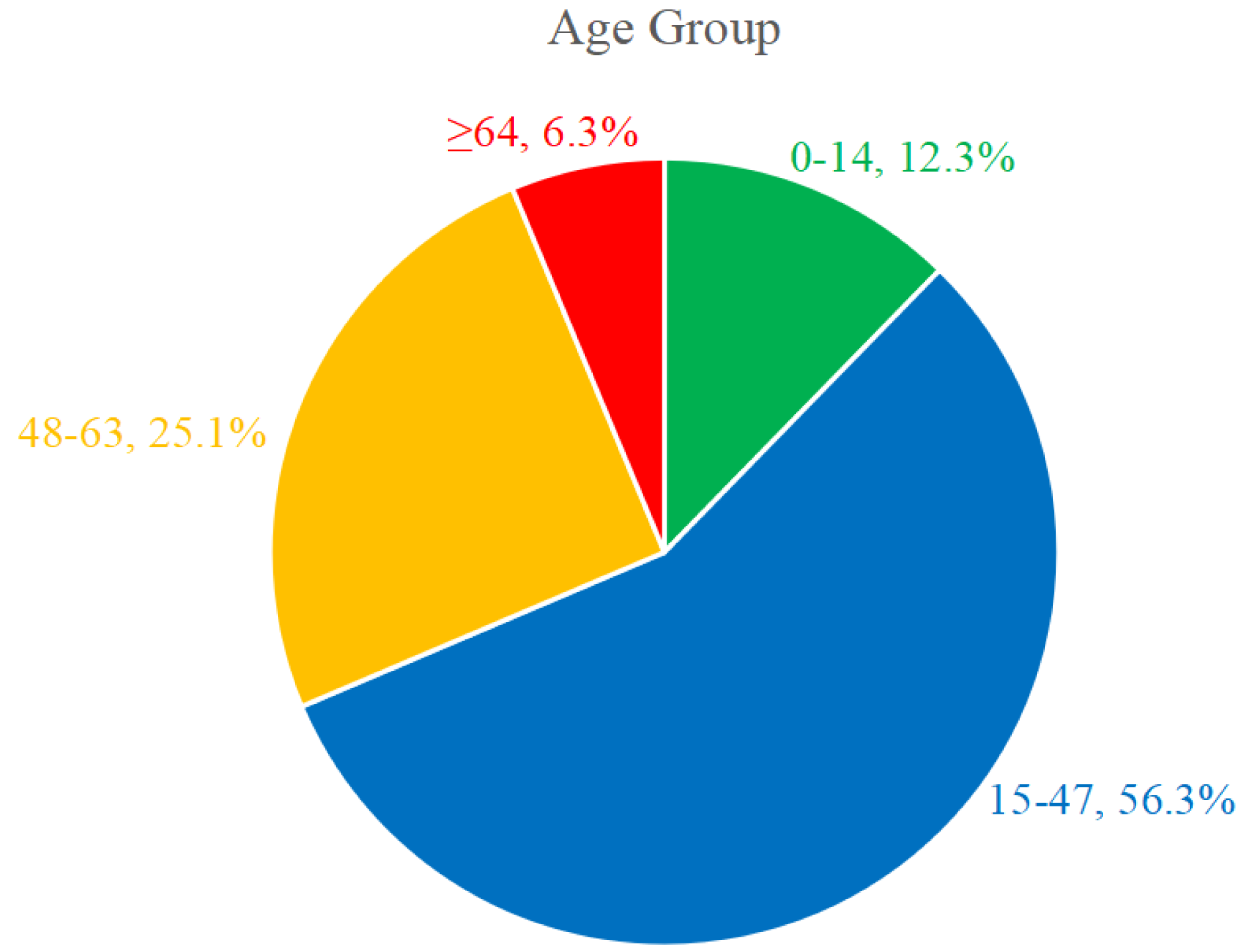

Supplement: Supplementary file 2 — Additional file 2: Figure S2. Constituent ratios of four age groups. The proportion of patients in the four age groups 0–14, 15–47, 48–63 and ≥ 64 years old. [file 12883_2020_1888_MOESM2_ESM.tif]

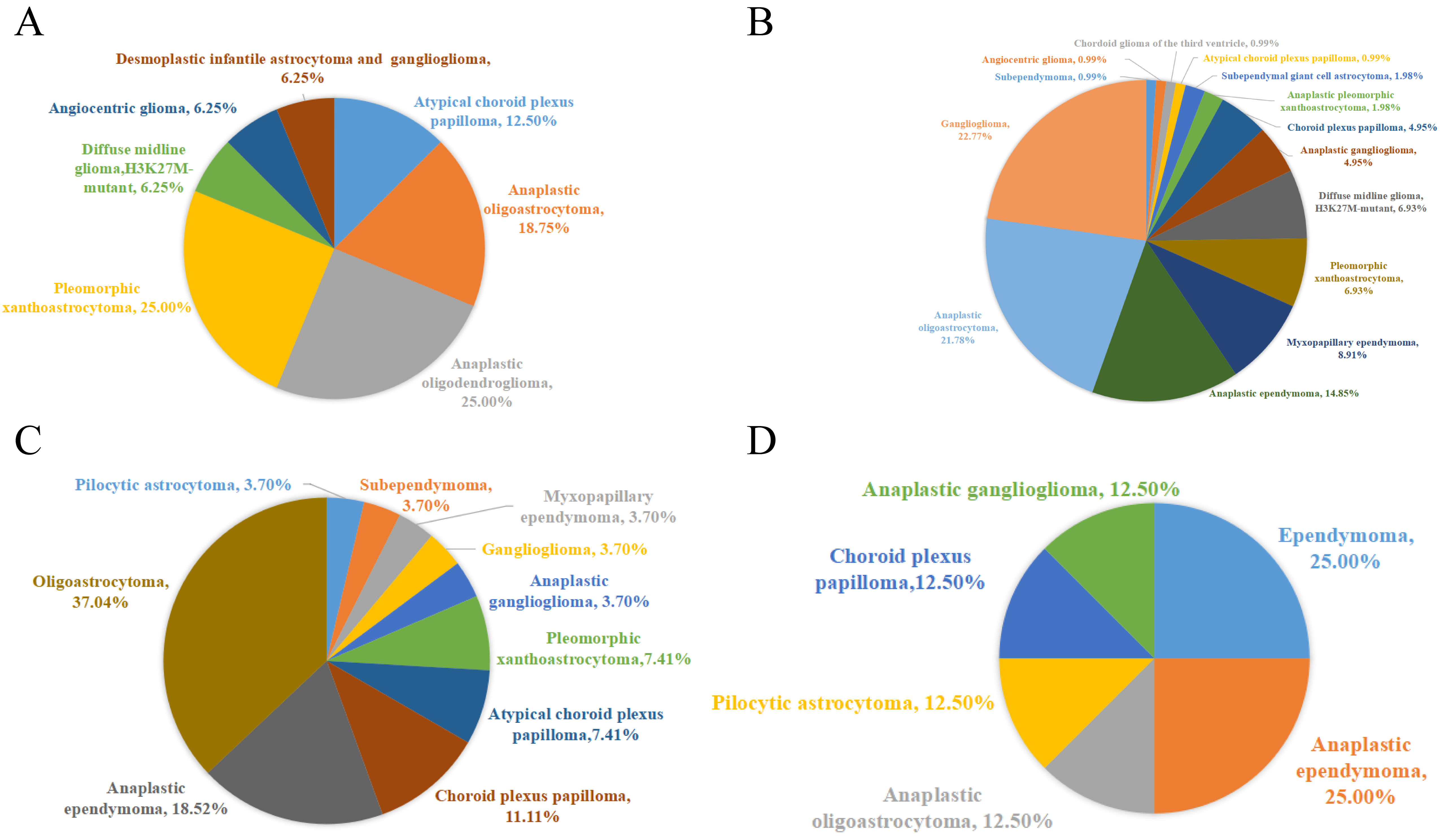

Supplement: Supplementary file 3 — Additional file 3: Figure S3. Distribution by Age groups of other histology. A: 0–14 years old. B: 15–47 years old. C: 48–63 years old. D: ≥64 years old. (subependymal giant cell astrocytoma, subependymoma, angiocentric glioma, chordoid glioma of the third ventricle, anaplastic ganglioglioma, desmoplastic infantile astrocytoma and ganglioglioma were not analyzed because the total number of patients was no more than three). [file 12883_2020_1888_MOESM3_ESM.tif]

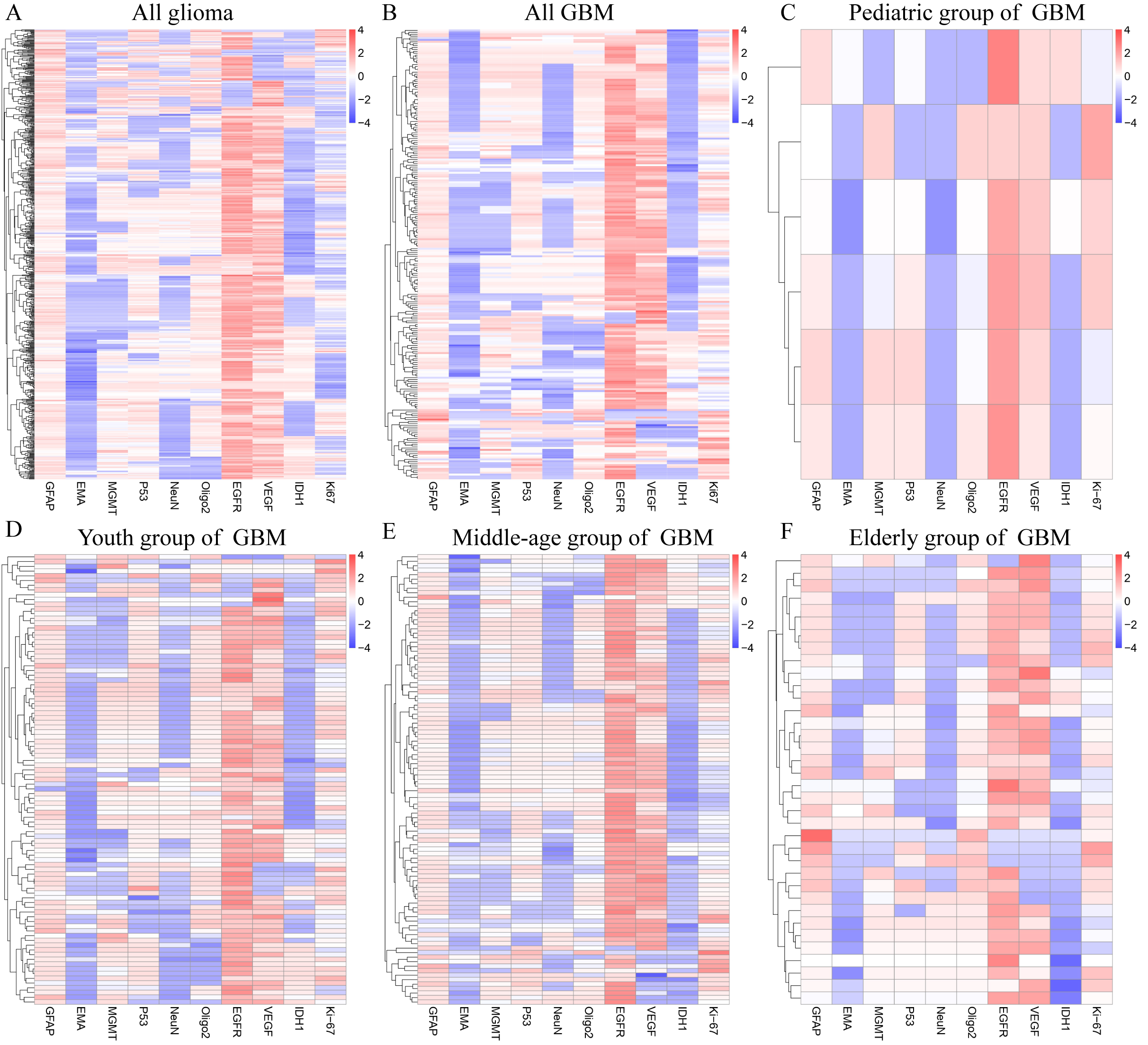

Supplement: Supplementary file 4 — Additional file 4: Figure S4. Heatmap of 10-gene signatures by gene expression subtype. Representative genes are shown for each subtype. A: Heatmap of all glioma. B: Heatmap of all GBM. C: Heatmap of pediatric group. D: Heatmap of youth old group. E: Heatmap of middle-age group. F: Heatmap of elderly group. [file 12883_2020_1888_MOESM4_ESM.tif]

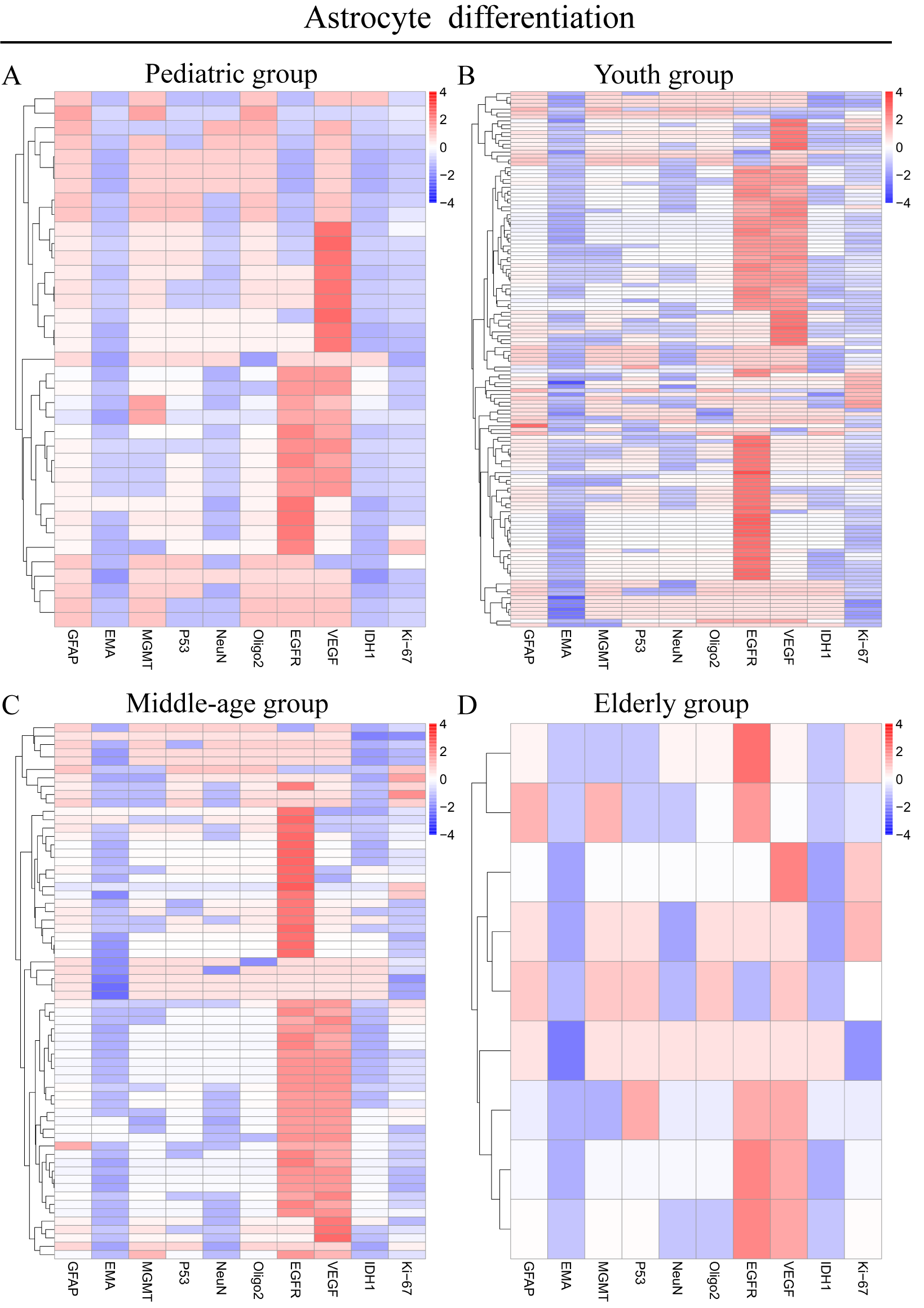

Supplement: Supplementary file 5 — Additional file 5: Figure S5. The heatmap of glioma derived from astrocyte differentiation (only including WHO grade I- III). A: Heatmap of pediatric group. B: Heatmap of youth group. C: Heatmap of middle-age group. D: Heatmap of elderly group. [file 12883_2020_1888_MOESM5_ESM.tif]

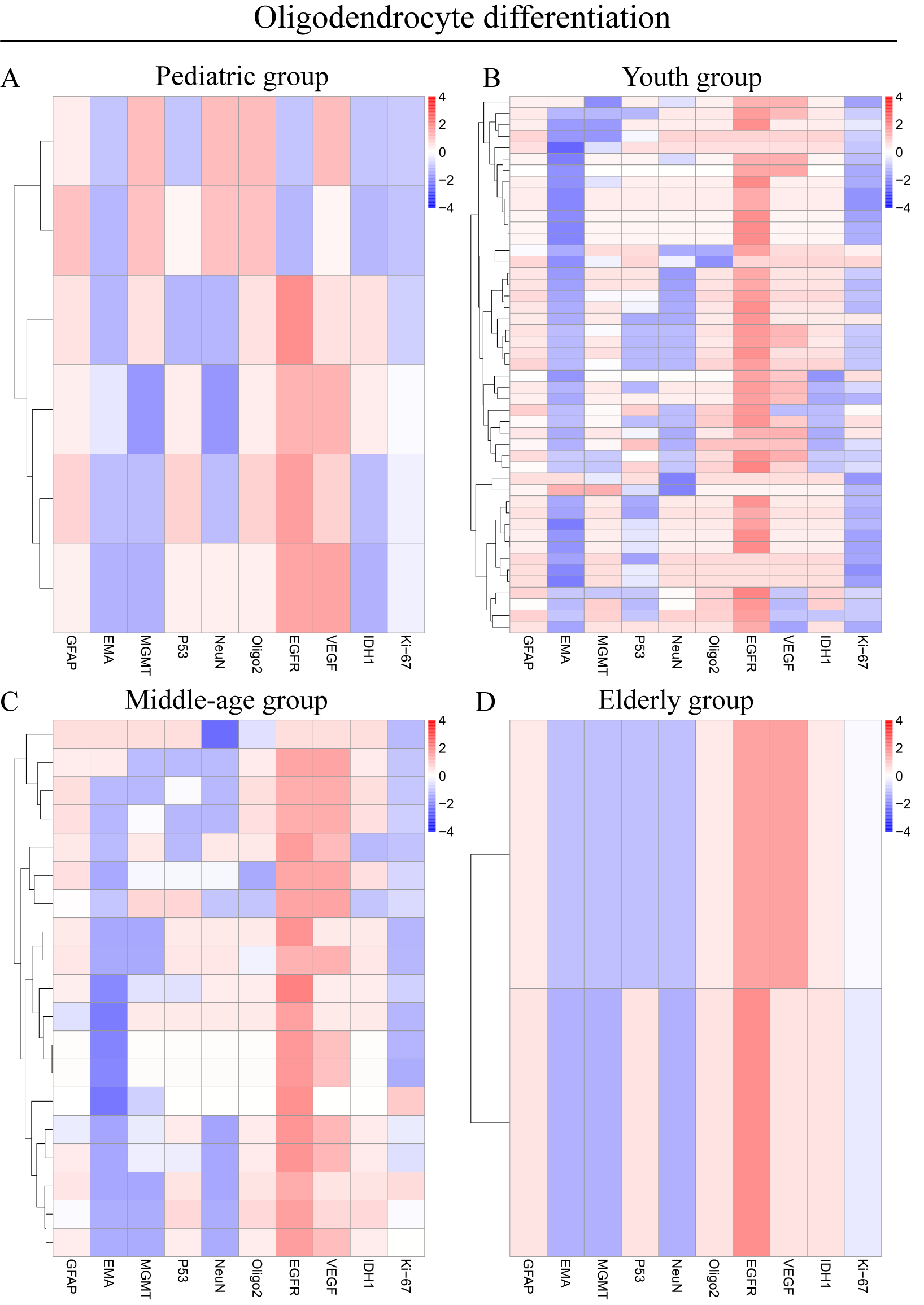

Supplement: Supplementary file 6 — Additional file 6: Figure S6. The heatmap of glioma derived from oligodendrocyte differentiation. A: Heatmap of pediatric group. B: Heatmap of youth group. C: Heatmap of middle-age group. D: Heatmap of elderly group. [file 12883_2020_1888_MOESM6_ESM.tif]

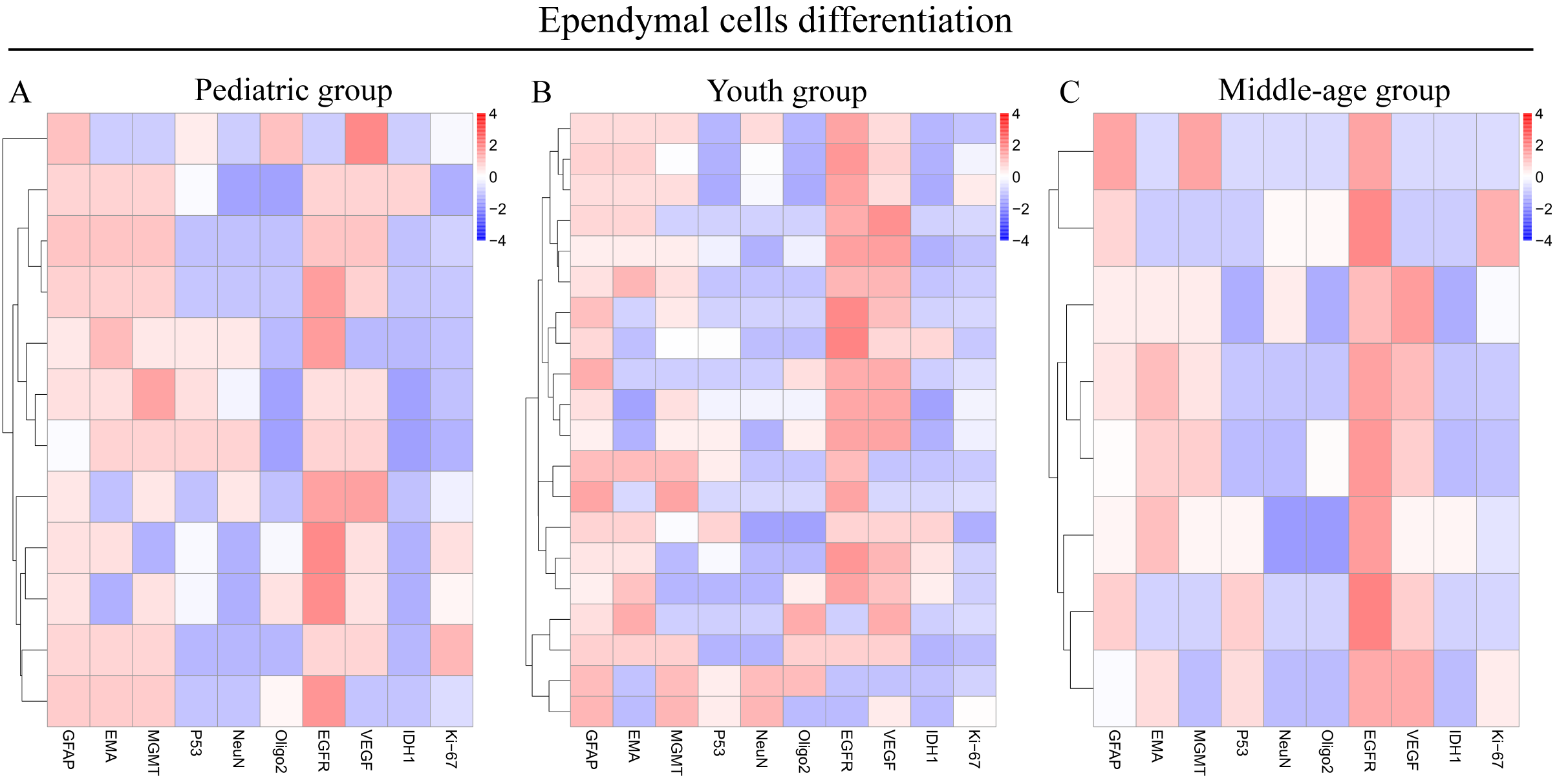

Supplement: Supplementary file 7 — Additional file 7: Figure S7. The heatmap of glioma derived from ependymal cells differentiation. A: Heatmap of pediatric group. B: Heatmap of youth group. C: Heatmap of middle-age group. D: Heatmap of elderly group. [file 12883_2020_1888_MOESM7_ESM.tif]

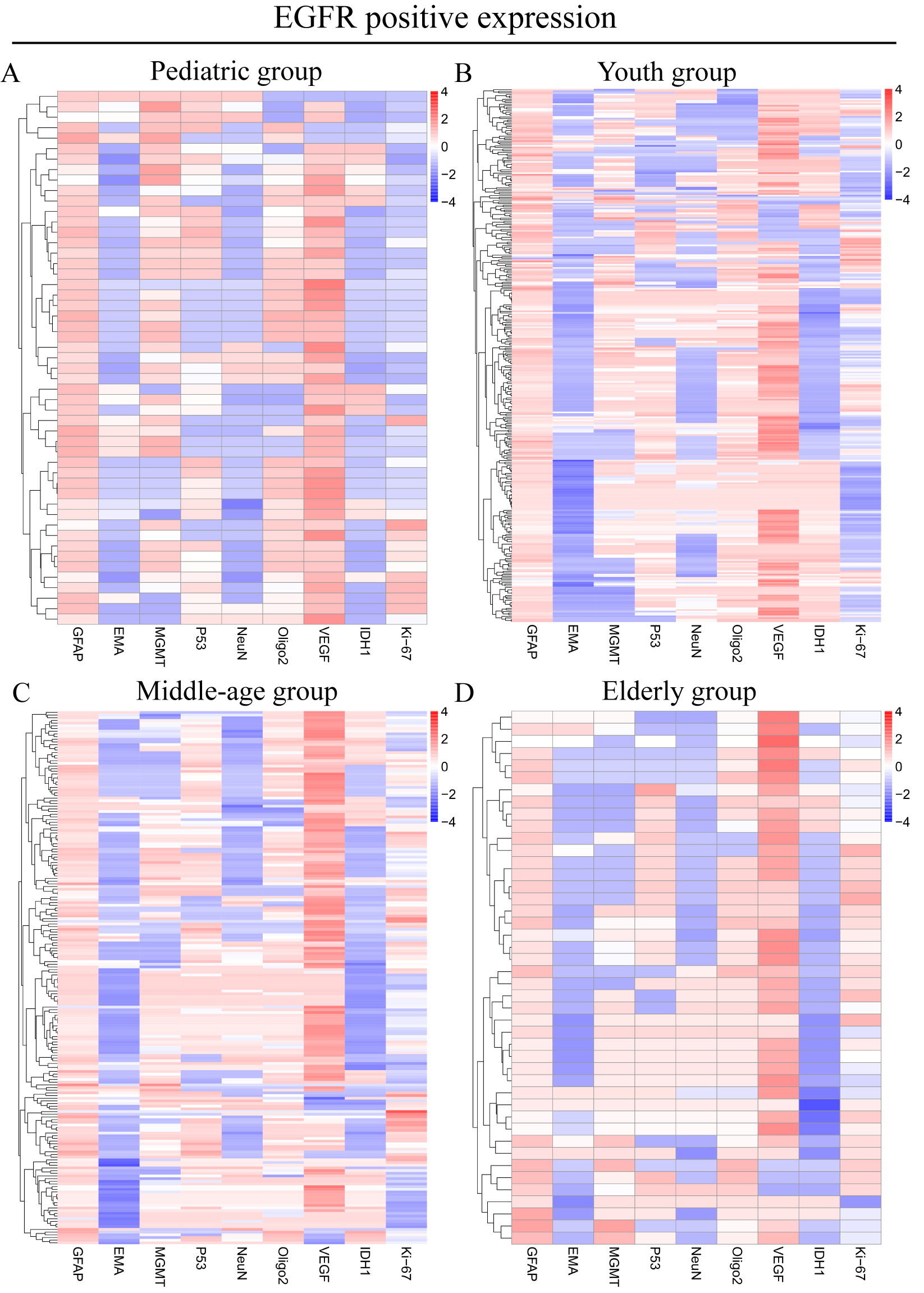

Supplement: Supplementary file 8 — Additional file 8: Figure S8. The heatmap of glioma with EGFR positive expression. A: Heatmap of pediatric group. B: Heatmap of youth group. C: Heatmap of middle-age group. D: Heatmap of elderly group. [file 12883_2020_1888_MOESM8_ESM.tif]

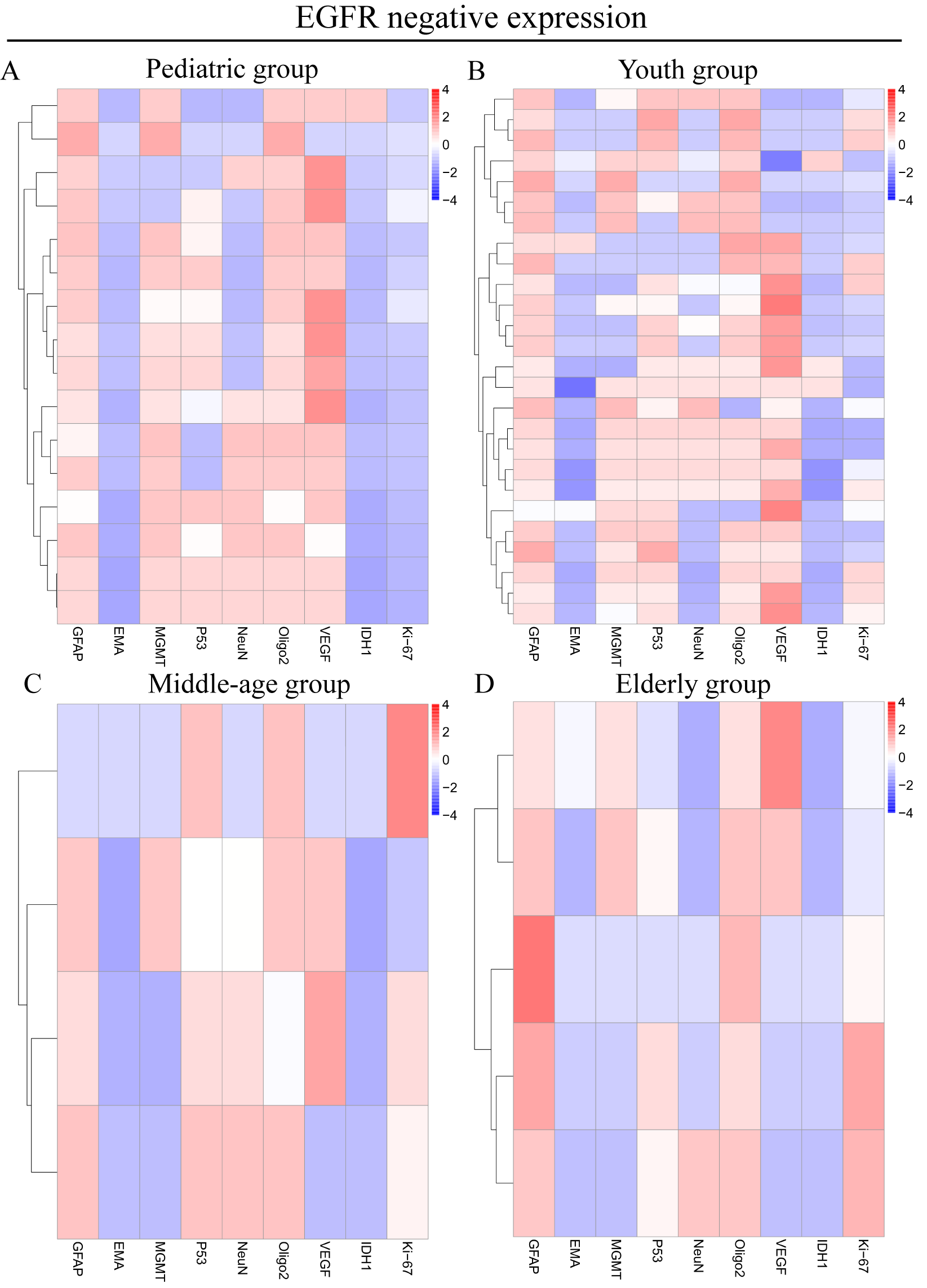

Supplement: Supplementary file 9 — Additional file 9: Figure S9. The heatmap of glioma with EGFR negative expression. A: Heatmap of pediatric group. B: Heatmap of youth group. C: Heatmap of middle-age group. D: Heatmap of elderly group. [file 12883_2020_1888_MOESM9_ESM.tif]

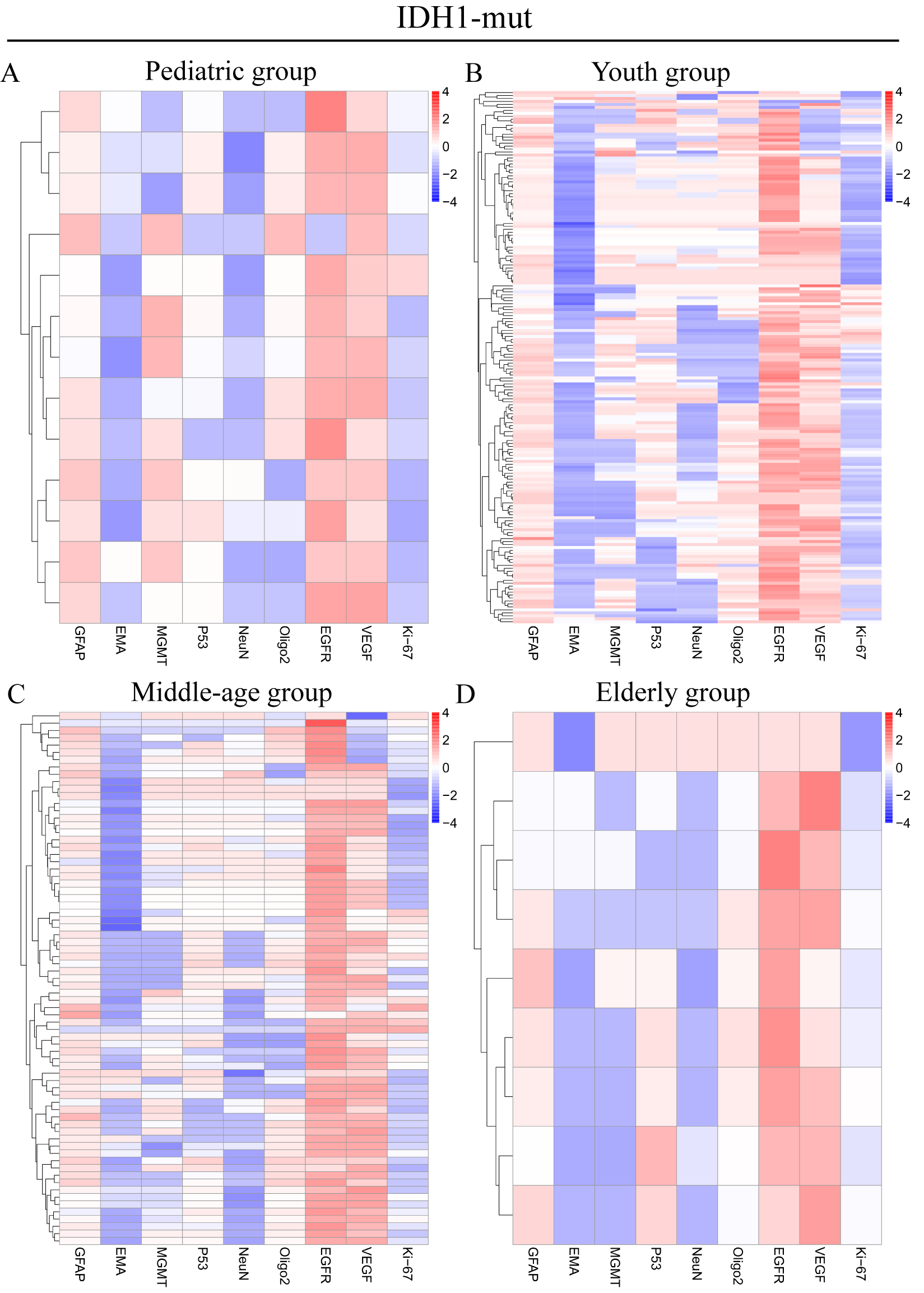

Supplement: Supplementary file 10 — Additional file 10: Figure S10. The heatmap of IDH1-mut glioma. A: Heatmap of pediatric group. B: Heatmap of youth group. C: Heatmap of middle-age group. [file 12883_2020_1888_MOESM10_ESM.tif]

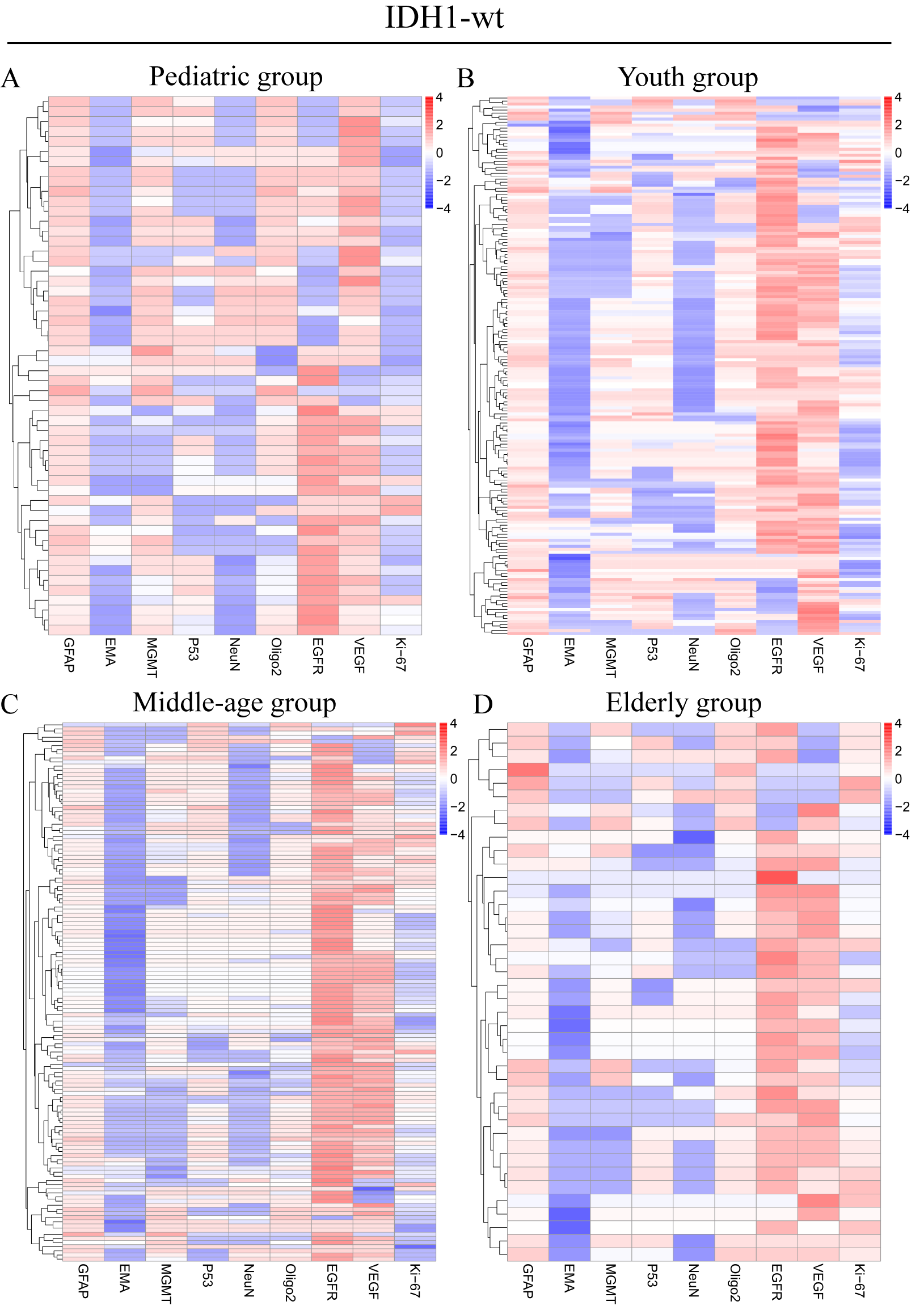

Supplement: Supplementary file 11 — Additional file 11: Figure S11. The heatmap of IDH1-wt glioma. A: Heatmap of pediatric group. B: Heatmap of youth group. C: Heatmap of middle-age group. [file 12883_2020_1888_MOESM11_ESM.tif]
